# Supplementary material for: Disruption of the Expression of the Placental Clock and Melatonin Genes in Preeclampsia
Source: Int J Mol Sci. 2023 Jan 25;24(3):2363. doi: 10.3390/ijms24032363 (PMC9917141; doi:10.3390/ijms24032363)
Supplement: Supplementary file 1 [file ijms-24-02363-s001.zip › ijms-2103641-supplementary.pdf]

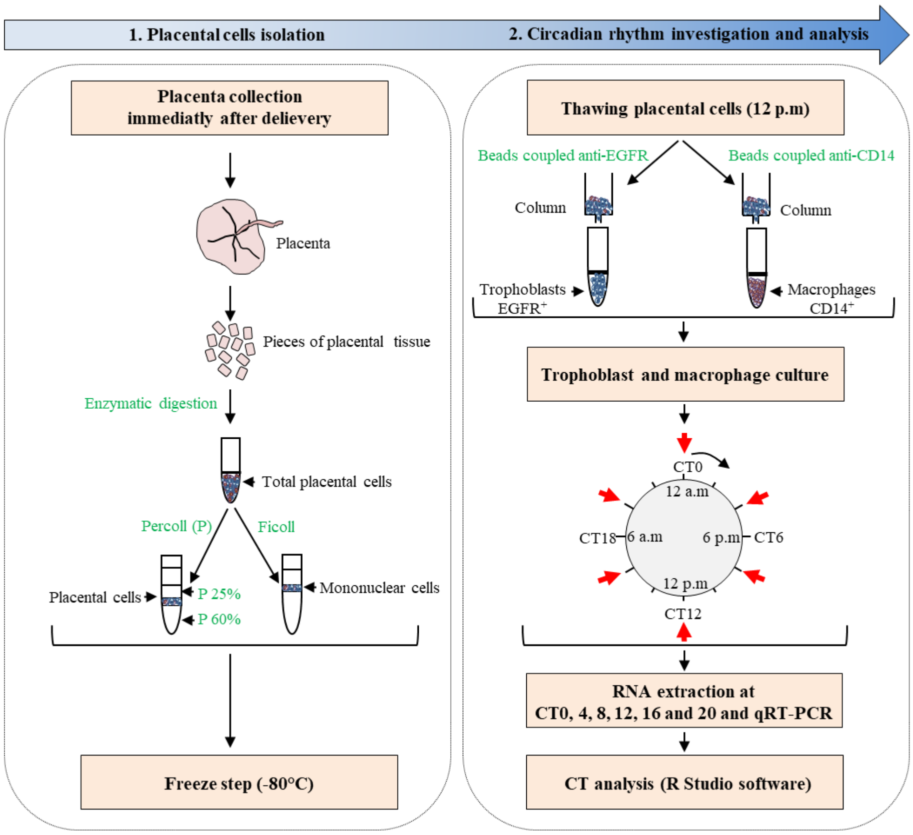

Supplemental figure 1. Schematic representation of the method used from the placental cell isolation to circadian rhythm investigation.

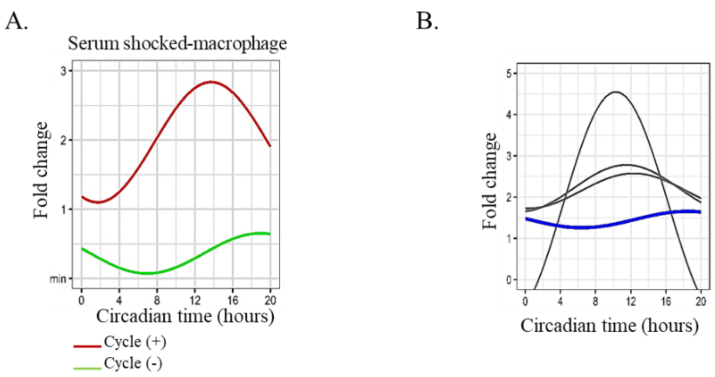

Supplemental figure 2. Macrophage and circadian rhythm

*(**A**) Macrophages from six healthy donors were synchronized with 50% FBS for 2 hours before CT0. The mean of the fold change of 6 biological replicates was represented in red and green for macrophages with (n=3) and without (n=3) cycles, respectively. Each sinusoid (in gray) represents the expression of the clock genes over time for each donor after adjustment of the values by the*

Cosinor model (mean of 3 technical replicates for each donor). The mean of the fold change of biological replicates was represented in blue. The statistical analysis was performed using the Cosinor function using R studio.

Supplemental Table 1. List of primers

Gene symbol	Forward primer (5′-3′)	Reverse primer (3′-5′)
ACBT	GGAAATCGTGCGTGACATTA	AGGAAGGAAGGCTGGAAGAG
BMAL1	AAACCAACTTTTCTATCAGACGATGAA	TCGGTCACATCCTACGACAAAC
CLOCK	AAGTTAGGGCTGAAAGACGACG	GAACTCCGAGAAGAGGCAGAAG
PER2	CGGAGTTAGAGATGGTGGAAGA	GGGACTGGAAAATGCTGAGTT
CRY2	CATGGCGACAGTCAGCAAAC	ACGTTTCCCACCACTGAGAG
NR1D1	AGAGCACCAGCAACATCACCAAGC	TTCTTGAAGCGACATTGCTGGCAG
MT2	AACTGCTGCGAGGCG	GGCGGTGGTGACGATG
AANAT	AATCAGAAGGGAACAGTA	TGGATGACAAATAGACAAG
ASMT	GGGCGTGTTTGACCTTCTCG	GCTCGCCCTCGGACCTGTAG

Supplemental Table 2. Rhythmic parameters (mesor, amplitude and acrophase) for macrophages co-cultured with trophoblasts for 1 day. A p value <0.05 for mesor, amplitude and acrophase implies a rhythmicity of the investigated gene

Gene	Mesor	CI 95%	*P*	Amplitude	CI 95%	*P*	Acrophase	CI 95%	*P*
BMAL1	1.64	(1.17; 2.11)	**<0.001**	1.3	(0.68; 2.00)	**<0.001**	16.99	(15.12; 18.87)	**<0.001**
CLOCK	1.26	(0.81; 1.97)	**<0.001**	1.39	(0.82; 1.97)	**<0.001**	13.08	(11.50; 14.66)	0.18
CRY2	3.07	(1.98; 4.17)	**<0.001**	1.87	(0.33; 3.41)	**0.02**	11.70	(8.55; 14.85)	0.85
PER2	0.91	(0.55; 1.27)	**<0.001**	0.83	(0.31; 1.33)	**0.001**	7.84	(5.48; 10.19)	**<0.001**
NR1D1	1.27	(0.90; 1.64)	**<0.001**	1.49	(0.96; 2.01)	**<0.001**	13.76	(12.41; 15.11)	**0.01**
